# Associations between personal apparent temperature exposures and asthma symptoms in children with asthma

**DOI:** 10.1371/journal.pone.0293603

**Published:** 2023-11-13

**Authors:** Linchen He, Shoshana Evans, Christina Norris, Karoline Barkjohn, Xiaoxing Cui, Zhen Li, Xiaojian Zhou, Feng Li, Yinping Zhang, Marilyn Black, Michael H. Bergin, Junfeng (Jim) Zhang

**Affiliations:** 1 Department of Community and Population Health, College of Health, Lehigh University, Bethlehem, Pennsylvania, United States of America; 2 Department of Epidemiology, Gillings School of Global Public Health, University of North Carolina at Chapel Hill, Chapel Hill, North Carolina, United States of America; 3 Department of Civil and Environmental Engineering, Duke University, Durham, North Carolina, United States of America; 4 Office of Research and Development, US Environmental Protection Agency, Research Triangle Park, North Carolina, United States of America; 5 Nicholas School of the Environment, Duke University, Durham, North Carolina, United States of America; 6 Department of Pediatrics, Shanghai General Hospital, Shanghai Jiao Tong University, Shanghai, China; 7 Department of Pulmonary Medicine, Shanghai Chest Hospital, Shanghai Jiao Tong University, Shanghai, China; 8 Department of Building Science, Tsinghua University, Beijing, China; 9 Beijing Key Laboratory of Indoor Air Quality Evaluation and Control, Beijing, China; 10 Underwriters Laboratories, Inc, Marietta, Georgia, United States of America; 11 Duke Global Health Institute, Duke University, Durham, North Carolina, United States of America; 12 Duke Kunshan University, Kunshan, Jiangsu Province, China; Balochistan University of Information Technology Engineering and Management Sciences, PAKISTAN

## Abstract

Ambient temperature and relative humidity can affect asthma symptoms. Apparent temperature is a measure of temperature perceived by humans that takes into account the effect of humidity. However, the potential link between personal exposures to apparent temperature and asthma symptoms has not been investigated. We conducted a panel study of 37 asthmatic children, aged 5–11 years, during an early spring season (average daily ambient temperature: 14°C, range: 7–18°C). Asthma symptoms were measured 4 times for each participant with a 2-week interval between consecutive measurements using the Childhood Asthma-Control Test (C-ACT). Average, minimum, and maximum personal apparent temperature exposures, apparent temperature exposure variability (TV), and average ambient temperature were calculated for the 12 hours, 24 hours, week, and 2 weeks prior to each visit. We found that a 10°C lower in 1-week and 2-week average & minimum personal apparent temperature exposures, TV, and average ambient temperature exposures were significantly associated with lower total C-ACT scores by up to 2.2, 1.4, 3.3, and 1.4 points, respectively, indicating worsened asthma symptoms. Our results support that personal apparent temperature exposure is potentially a stronger driver than ambient temperature exposures for the variability in asthma symptom scores. Maintaining a proper personal apparent temperature exposure could be an effective strategy for personalized asthma management.

## 1. Introduction

Asthma is a chronic and noncommunicable disease that impacts the respiratory system. Over the past 20 years, asthma prevalence has been increasing in many regions of the world [1, 2]. As an uncurable and heterogeneous disease, it is imperative to develop tailored intervention strategies for asthma treatment and management [3].

Triggers for asthma include allergens, air pollutants, tobacco smoke, and physical activity [4]. In addition, breathing cold air is a risk factor for asthma exacerbation [5]. It has been reported that, in the cold season, lower ambient temperature was associated with a higher relative risk of asthma-related hospital admissions [6–8] and emergency department visits [9]. The potential underlying biological mechanisms include that breathing cold air can induce bronchoconstriction, leading to airway inflammation and airflow limitation [4, 10, 11]. In addition to temperature, humidity could also affect asthma. Higher ambient relative humidity has been associated with higher emergency department visits for asthma exacerbation [12]. Breathing air with low humidity could lead to a higher risk of asthma exacerbation by drying the mucosal membrane along the airway [13].

Apparent temperature is a measure of temperature perceived by humans that takes into account the effect of humidity [14]. Considering that both temperature and humidity can strongly affect asthma, apparent temperature, as a single meteorological factor, could be useful in personalized asthma management. However, to the best of our knowledge, no study has investigated the effects of apparent temperature exposure on asthma symptoms.

In this study, we used data collected from a cohort of 37 asthmatic children and examined the associations of asthma symptom scores (Childhood Asthma-Control Test [C-ACT]) with average, minimum, and maximum personal apparent temperature exposures, personal apparent temperature exposure variability (TV), and average ambient temperature exposures. All the exposure metrics were calculated during the 12 hours, 24 hours, 1 week, and 2 weeks prior to the clinical visits. We hypothesized that lower personal apparent temperature exposure is associated with lower C-ACT scores, indicating worsened asthma symptoms. This study aims to explore three questions, namely: (1) what are the impacts of colder air exposure on asthma symptoms, (2) do short-term (12-hour or 24-hour) and long-term (1-week or 2-week) apparent temperature exposures affect asthma symptoms differently, and (3) is personal apparent temperature exposure a stronger driver than ambient temperature for the variability in the asthma symptom scores.

## 2. Methods

### 2.1. Study participants

Thirty-seven children aged 5–11 years old with mild or moderate asthma were recruited from the Department of Pediatrics at Shanghai General Hospital, Shanghai, China. Children with chronic diseases other than asthma were excluded from this study. Oral assent and written consent were obtained from the asthmatic children and their caregivers, respectively. The current analysis leveraged the data from a previous study designed for investigating the pulmonary health impacts of air pollution exposure [15–17]. The authors had access to information that could identify individual participants during the data collection stage, while all the identifiable information was removed from the data after it had been collected.

### 2.2. Measurements of health outcomes

Between February 14 and April 14, 2017, each participant was clinically assessed four times at two-week intervals. Efforts were made to schedule the four visits at the same time of the day. During each visit, each child filled out the Childhood Asthma Control Test (C-ACT) with their caregivers. The C-ACT, developed by GlaxoSmithKline (GSK) and validated in children 4 to 11 years old, has been widely used in pediatric asthma management [18]. Compared to conventional spirometry lung function, C-ACT could provide a subjective assessment of asthma control and is particularly useful for younger children, due to their limited ability to cooperate during a spirometry test. The C-ACT includes seven questions in total. The first four questions were answered by children on asthma control, limitation of physical activities, coughing, and waking up at night. The last three questions were answered by caregivers on children’s daytime symptoms, daytime wheezing, and waking up at night during the past four weeks. A score ranging from 0–3 was assigned by children for each of the first four questions, and a score ranging from 0–5 was assigned by caregivers for each of the last three questions [19]. A higher score for individual questions indicates improved asthma symptoms, and a higher total C-ACT score means better control of asthma symptoms. A detailed description of the seven questions is shown in S1 Table.

### 2.3. Apparent temperature exposure assessment

Temperature and relative humidity were continuously measured in participants’ bedrooms and outside a window of their homes, using a sensor box equipped with an Sensirion AG sensor (SHT15). The sensor box generated hourly averages for each of the environmental factors. The sensor box was field-calibrated before and after the current study. In addition, hourly averages of ambient temperature and relative humidity were obtained from the governmental environmental monitoring station closest to the research hospital where participants were recruited (about 9 kilometers away). We calculated 12-hour, 24-hour, 1-week, and 2-week average ambient temperature exposures prior to each clinical visit. Two assumptions were made to estimate the temperature and relative humidity levels in other indoor microenvironments (e.g., classroom) where no actual environmental measurements were obtained. First, we assumed that there were linear relationships between indoor and outdoor temperature and relative humidity levels. Second, those linear relationships crossing all participants’ homes were similar to that in the other indoor microenvironments at the same hour of a day. Specifically, we constructed 24 linear regression models, in which indoor temperature measured in participants’ bedrooms was the dependent variable, and outdoor temperature measured outside a window of the home was the independent variable. Each of these regression models indicates the relationship between indoor and outdoor temperatures for one hour of a day. Finally, based on these models, we used ambient temperature levels as the independent variable to predict temperature levels in other microenvironments for each hour of a day.

Using all the measured and calculated temperature and relative humidity data, apparent temperature was calculated for each of the microenvironments using the equations developed by Rothfusz [20] and Steadman [14, 21] (see S2 Table). All the microenvironmental levels were coupled with detailed time-activity data reported by the participants to calculate 12-hour, 24-hour, 1-week, and 2-week average personal apparent temperature exposure prior to each clinical visit. In addition, we calculated 12-hour and 24-hour minimum apparent temperature exposures as the lowest hourly exposure within the 12-hour or 24-hour period prior to each visit, respectively. The 1-week and 2-week minimum exposures were calculated as the average of 7 and 14 daily minimum personal exposures prior to each visit, respectively. We used the same method to calculate maximum personal apparent temperature exposures. In addition, we calculated 12-hour and 24-hour personal apparent temperature exposure variability (TV) as the standard deviation of the minimum and maximum hourly exposures 12 hours and 24 hours prior to each visit, respectively. Similarly, 1-week and 2-week TV were calculated as the standard deviation of all the daily maximum and minimum exposures within 7 days and 14 days, respectively. Detailed assessments of personal apparent temperature exposures and TV are shown in S1 Fig in the Supplemental Information. By conducting the current exposure assessment, we aim to explore (1) whether short-term (12-hour or 24-hour) and long-term (1-week or 2-week) apparent temperature exposures affect asthma symptoms differently and (2) whether personal apparent temperature exposure is a stronger driver than ambient temperature for the variability in the asthma symptom scores.

Fine particulate matter (PM_2.5_) and ozone (O_3_) were also measured by the air sensor box used in this study [22, 23]. We further calculated personal exposures to PM_2.5_ and O_3_, as they might affect asthma symptoms [16, 17, 24]. The detailed method for personal air pollution exposure assessment has been published previously [25], and the results are shown in S3 Table.

### 2.4. Statistical analyses

First, we used linear mixed-effect regression (LMER) models to investigate the cross-sectional associations between C-ACT scores and personal apparent temperature exposures (average, minimum, maximum, and variability) 12 hours, 24 hours, 1 week, or 2 weeks prior to clinical visits. In these models, each individual or total C-ACT score was the dependent variable, and one of the personal apparent temperature exposures was the independent variable. We controlled for fixed-effects covariates including baseline eosinophil count (continuous), inhaled corticosteroid usage status (yes/no), respiratory tract infection status (yes/no), asthma exacerbation status (yes/no), age (continuous), sex (male/female), and personal PM_2.5_ and O_3_ exposures (continuous) averaged over the same period as the main apparent temperature exposure. Subject ID was the random-effect variable. From the model output, we calculated change (and 95% confidence interval) in C-ACT scores associated with a 10°C lower in personal apparent temperature exposures or a 10°C higher in TV. By conducting these analyses, we aim to explore whether short-term (12-hour or 24-hour) and long-term (1-week or 2-week) apparent temperature exposures affect asthma symptoms differently.

Second, we used LMER models to examine the associations between average ambient temperature and C-ACT scores, controlling for the covariate structures as described in the first set of models. We further controlled for average ambient relative humidity over the same period of ambient temperature exposures as an additional fixed-effect variable. We conducted this analysis to determine whether personal apparent temperature exposure is a stronger driver than the ambient temperature for the variability in the asthma symptom scores.

Lastly, we explored whether the associations of total C-ACT score with personal apparent temperature exposures and TV were moderated by sex by adding an interaction term between sex and each of the exposures. In addition, to further investigate how sex moderated our main associations, we used the LMER models to re-examine the main associations after stratifying by sex (male versus female) following the same covariate structure as described in the first set of models.

We also conducted a sensitivity analysis by re-examining the associations described in the first set of models in a sub-dataset, excluding measurements from children who had physician-diagnosed asthma exacerbation during the 2 weeks prior to the clinical visit. All analyses of associations were conducted using *lme4* and *lmeTest* packages in R software (version 4.0.2) [26, 27]. We examined the repeated measure correlations among personal apparent temperature exposures, TV, and average ambient temperature exposure using the *rmcorr* package [28]. A P-value of 0.05 was set as the cut point for statistical significance. Detailed descriptions of the statistical analyses and detailed model results (S4 and S5 Tables) are provided in the Supplemental Information.

## 3. Results

### 3.1. Participant characteristics

Detailed characteristics of the participants are listed in Table 1. Of the 37 participants, 16 (43%) were female, 26 (70%) completed all four visits, 7 (19%) completed three visits, and 4 (11%) completed two visits. The mean±SD age of all participants was 7.1±1.6 years. From a total of 133 C-ACT measurements, inhaled corticosteroids were used prior to 28 (21%) measurements, respiratory tract infection-like symptoms were reported prior to 55 (41%) measurements, and asthma exacerbation during the two weeks prior to clinical visits was reported prior to 7 (5%) measurements.

**Table 1:** Characteristics of participants.

Subject Characteristics	Value
**Number of participants, N**	37
Age, mean ± SD [range] (years)	7.1 ± 1.6 [5–11]
Female, No. (%)	16 (43%)
Height, mean ± SD [range] (cm)	129 ± 10.6 [110–157]
Weight, mean ± SD [range] (Kg)	29.4 ± 9.3 [19–55]
Baseline Blood eosinophil count, mean ± SD [range] (/μL)	327 ± 183 [80–850]
Baseline eosinophilic inflammation, No. (%)	8 (22)
**Total number of visits, N**	133
Inhaled corticosteroids usage, No. (%)	28 (21)
Respiratory tract infection, No. (%)	55 (41)
Asthma exacerbation, No. (%)	7 (5)

### 3.2 Personal apparent temperature exposures and C-ACT scores

The average, minimum, and maximum personal apparent temperature exposures, apparent temperature exposure variability (TV), and average ambient temperature over the 12 hours, 24 hours, 1 week, and 2 weeks prior to each visit are shown in Table 2. The repeated measures correlations among personal apparent temperature exposures, TV, and average ambient temperature exposure are shown in S6 Table. As expected, we found that average, minimum, and maximum personal apparent temperature exposures and average ambient temperature exposure were positively associated with each other, while none of them was strongly associated with TV. Statistical summaries of the individual and total C-ACT scores across all visits are shown in Table 3. Of the 133 visits, 7 of them reported a total C-ACT score ≤ 19, which is a sign that the child’s asthma is not well controlled at that time [18].

**Table 2:** Statistical summaries of personal apparent temperature exposures.

	Mean ± SD	Median [IQR]	Range
**Average Personal Apparent Temperature Exposure** (°C)			
12-hour	18.2 ± 2.6	17.8 [3.5]	12.7–24.3
24-hour	18.3 ± 2.5	17.9 [3.4]	12.7–23.8
1-week	18.0 ± 2.4	17.6 [2.8]	12.8–24.9
2-week	17.6 ± 1.9	17.3 [2.5]	12.9–22.8
**Minimum Personal Apparent Temperature Exposure** (°C)			
12-hour	14.9 ± 4.4	15.4 [5.8]	1.8–22.4
24-hour	14.0 ± 4.1	14.6 [5.3]	1.8–21.1
1-week	13.9 ± 3.4	14.2 [4.9]	4.4–20.2
2-week	13.2 ± 3.3	13.7 [4.6]	4.2–19.2
**Maximum Personal Apparent Temperature Exposure** (°C)			
12-hour	20.0 ± 2.9	19.0 [3.3]	13.3–38.1
24-hour	20.5 ± 3.0	19.1 [3.3]	16.6–38.1
1-week	20.1 ± 2.4	19.3 [2.9]	16.7–29.2
2-week	19.7 ± 1.8	19.2 [2.1]	16.7–25.8
**Personal Apparent Temperature Exposure Variability** (°C)			
12-hour	3.6 ± 2.5	3.2 [3.7]	0.2–12.1
24-hour	4.6 ± 2.3	4.4 [3.1]	0.6–12.1
1-week	3.8 ± 1.4	3.5 [1.7]	1.4–7.8
2-week	4.0 ± 1.4	3.7 [1.9]	1.5–7.6
**Average Ambient Temperature Exposures** (°C)			
12-hour	12.3 ± 3.9	11.2 [5.0]	3.5–21.8
24-hour	12.5 ± 3.8	11.8 [5.4]	4.2–21.4
1-week	12.1 ± 3.8	10.8 [4.5]	5.3–21.7
2-week	11.5 ± 3.0	10.5 [4.4]	5.8–18.2

**Table 3:** Statistical summaries of C-ACT scores.

	Mean ± SD	Median [IQR]	Range
**Individual Scores: reported by child** (Ranging from 0–3 with 3 indicating better symptoms)			
Asthma control	2.6 ± 0.5	3 [1]	1–3
Limitation of physical activities	2.5 ± 0.7	3 [1]	0–3
Coughing	2.3 ± 0.8	2 [1]	0–3
Waking up at night	2.8 ± 0.4	3 [0]	2–3
**Individual Scores: reported by caregiver** (Ranging from 0–5 with 5 indicating better symptoms)			
Daytime asthma symptoms	4.7 ± 0.7	5 [1]	0–5
Wheezing	4.8 ± 0.5	5 [0]	2–5
Waking up at night	4.9 ± 0.4	5 [0]	3–5
**Total C-ACT Scores**	24.6 ± 2.4	25 [3]	16–27

### 3.3 The associations of C-ACT scores with personal apparent temperature exposures and TV

**Fig 1:**
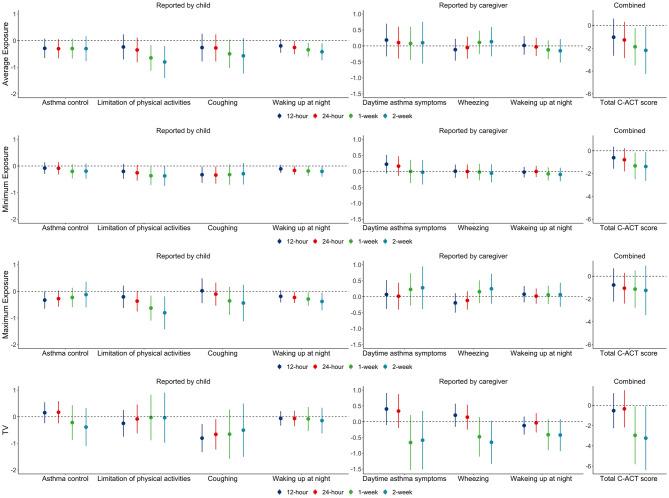
The change in C-ACT scores associated with a 10°C lower in personal apparent temperature exposures or a 10°C higher in TV.

The associations of C-ACT scores with average, minimum, and maximum personal apparent temperature exposures and TV are shown in Fig 1. The results indicate that a 10°C lower in 1-week and 2-week average apparent temperature exposures were significantly associated with changes in total C-ACT scores by -1.9 (-3.5 to -0.2) and -2.2 (-4.3 to -0.1), respectively. Similarly, a 10°C lower in 1-week and 2-week minimum apparent temperature exposures were significantly associated with changes in total C-ACT scores by -1.3 (-2.5 to -0.16) and -1.4 (-2.5 to -0.1), respectively. In addition, a 10°C lower 12-hour, 24-hour, 1-week, and 2-week maximum apparent temperature exposures were nonsignificantly associated with lower total C-ACT scores by -0.8 (-2.2 to 0.7), -1.1 (-2.4 to 0.3), -1.1 (-2.8 to 0.5), and -1.2 (-3.4 to 0.9), respectively. We found that a 10°C higher in 1-week and 2-week TV were significantly associated with changes in total C-ACT scores by -3.0 (-5.8 to -0.1) and -3.2 (-6.3 to -0.1), respectively. Lower personal apparent temperature exposures were significantly or borderline significantly associated with lower individual scores reported by the child. We did not find any significant associations between apparent temperature exposures and individual C-ACT scores reported by the caregiver.

### 3.4 The associations between C-ACT scores and average ambient temperature

**Fig 2:**
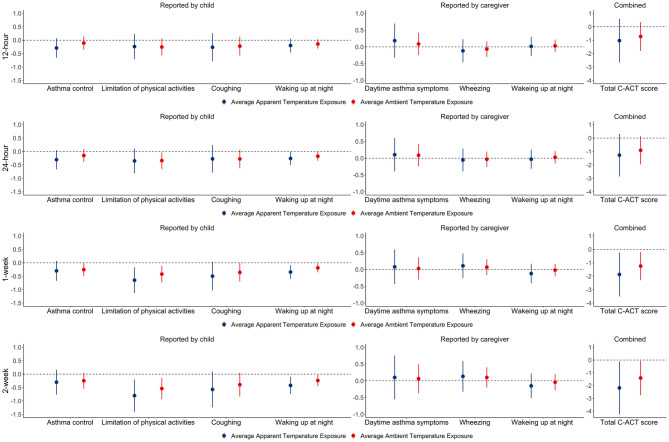
The change in C-ACT scores associated with a 10°C lower in average personal apparent temperature exposures and average ambient temperature exposure.

The associations between C-ACT scores and average ambient temperature are shown in Fig 2. We found that a 10°C lower in 1-week and 2-week average ambient temperature exposure (red line in Fig 2) was significantly associated with changes in total C-ACT score of -1.2 (-2.3 to -0.2) and -1.4 (-2.8 to -0.1), respectively. In addition, ambient temperature exposures were also adversely associated with individual C-ACT scores reported by the child, while not with scores reported by the caregiver. Furthermore, we found that, compared with average ambient temperature exposures, average personal apparent temperature exposures had stronger adverse effects on total C-ACT scores and individual C-ACT scores reported by the child. For example, a 10°C lower in 1-week and 2-week average apparent temperature exposure was associated with changes in total C-ACT scores by -1.9 (-3.5 to -0.2) and -2.2 (-4.3 to -0.1), respectively.

### 3.5 Effect modification

The interactions of sex with personal apparent temperature exposures and TV were assessed to explore whether the associations between total C-ACT score and temperature exposures were modified by sex. As shown in S7 Table, we found significant interactions between sex and 2-week minimum apparent temperature exposure and between sex and 2-week maximum apparent temperature exposure. To further explore the sex by temperature interaction effects, we performed sex-stratified analyses. The results show a significant association of total C-ACT score with apparent temperature exposures and with TV, respectively, in girls only (S2 Fig).

### 3.6 Sensitivity analysis

We re-examined the main associations in a sub-dataset, excluding measurements from participants who had asthma exacerbation during the 2 weeks prior to the clinical visit. As shown in S3 Fig, little difference was found in the associations of C-ACT scores with personal apparent temperature exposures and TV, in terms of statistical significance and effect size. We only found that the associations of the total C-ACT score with 1-week and 2-week average apparent temperature exposures were changed from statistically significant to borderline significant.

## 4. Discussion

Temperature and humidity have been widely reported to be able to affect asthma. Apparent temperature, as a single meteorological factor that takes into account both temperature and humidity, is firstly associated with C-ACT scores in this study. The results mainly show that lower 1-week and 2-week personal apparent temperature exposures (average, minimum, and TV) were significantly associated with lower total and individual C-ACT scores reported by the child. The results suggest the importance of maintaining a proper apparent temperature exposure as a strategy for pediatric asthma management.

As shown in Fig 1, we found that lower average and minimum personal apparent temperature exposures and higher TV were significantly associated with lower total C-ACT scores and individual scores reported by the child. The results are supported by previous epidemiological studies finding that higher asthma-related hospital admission was associated with lower daily mean ambient temperature [6, 29, 30], minimum ambient temperature [31, 32], and TV [33, 34]. In addition, we found that the adverse impacts of personal apparent temperature exposures on total C-ACT scores were mainly driven by the reduction in individual C-ACT scores reported by the child. However, we did not find any significant associations between apparent temperature exposures and individual C-ACT scores reported by the caregiver. The results suggest that children were more sensitive to changes in apparent temperature exposures than what was reported by their caregivers for the children’s asthma symptoms.

In this study, we explored whether short-term (12-hour or 24-hour) and long-term (1-week or 2-week) apparent temperature exposures affect asthma symptoms differently. We found that only long-term (i.e., 1-week and/or 2-week) average & minimum apparent temperature exposures and TV were significantly associated with total C-ACT scores, indicating worsened asthma control. In addition, compared to short-term exposures (i.e., 12-hour and 24-hour), a 10°C lower in long-term (i.e., 1-week and 2-week) apparent temperature exposures and TV were associated with larger drops in total C-ACT scores. These results were partially supported by a previous study reporting that, compared with 2-day average exposure, 2-week average ambient temperature exposure was more strongly associated with emergency department admissions for childhood asthma in the cold season [35]. Similar lagged effects of cold ambient temperature exposures on emergency department admissions for asthma have also been reported for adults [36]. The previous results along with our current findings suggest a lagged effect of colder temperature exposure on asthma symptoms. However, the underlying biological mechanism is still poorly understood and needs to be further explored. It has been reported that breathing cold air can adversely affect asthma symptoms by inducing bronchoconstriction, which further leads to airway inflammation and airflow limitation [4, 10, 11]. Our results led us to hypothesize that the adverse effects of short-term exposure to colder temperature on asthma symptoms are reversible or can be mitigated after asthmatic patients move to warmer environments. If this hypothesis holds true, it further suggests the importance of maintaining proper average personal exposure to apparent temperature for better asthma control.

The second question we explored is whether personal apparent temperature exposure is a stronger driver than ambient temperature exposure for the variability in the asthma symptom scores. Due to the positive correlations between average personal apparent temperature exposures and average ambient temperature for all the time scales (see S6 Table), they were similarly associated with C-ACT scores (see Fig 2). However, we found that, compared to the ambient temperature exposure, the personal apparent temperature exposures had stronger effects on total C-ACT scores. For example, a 10°C lower in 1-week and 2-week average apparent temperature exposure was associated with a lower total C-ACT score by -1.9 (-3.5 to -0.2) and -2.2 (-4.3 to -0.1), respectively. However, a 10°C lower in 1-week and 2-week average ambient temperature exposure was associated with a lower total C-ACT score only by -1.2 (-2.3 to -0.2) and -1.4 (-2.8 to -0.1), respectively. The impacts of ambient temperature and relative humidity exposures on asthma have often been investigated in previous studies, while not taking into account the effects of indoor exposure. It has been widely reported that people spend more than 80% of their time indoors. Indoor temperature and relative humidity could be dramatically different from ambient conditions, especially during the cold and hot seasons, when indoor heating or air conditioning is used. Hence, the personal exposure assessment method, as a strength, used in this study could provide a more accurate estimate of how temperature affects asthma symptoms. In addition, our results further suggest that, compared to ambient temperature exposure, personal apparent temperature exposure could be a stronger predictor for asthma symptom scores and a better exposure metric in personalized asthma management. More studies with a larger sample size and more asthma-related health outcomes are recommended to confirm our findings.

The minimal clinically important difference in adulthood Asthma Control Test scores is 3 points [37]. Although few studies have reported that a 2-point change could be a minimally important difference in C-ACT, it is not fully established yet [38, 39]. In this study, we found that a 10°C lower in 2-week average apparent temperature exposure and TV was associated with a lower total C-ACT score by 2.2 and 3.2 points, respectively, which indicate a potential clinically important difference in C-ACT. Our results indicate the importance of maintaining a proper apparent temperature exposure, by approaches like using indoor air conditioning and a humidifier during the cold season as a strategy for pediatric asthma management.

In this study, we examined whether the associations of total C-ACT scores with personal apparent temperature exposures and TV were modified by sex. We found significant interactions of sex with 2-week minimum and maximum apparent temperature exposures (see S7 Table). In addition, the results of stratification analyses show that asthma symptom scores were significantly associated with personal apparent temperature exposures and TV only in girls (see S2 Fig). The current results are inconsistent with a previous study finding that higher ambient temperature variation was significantly associated with higher relative risk of pediatric asthma only in males [40]. The previous and current results suggest that the impacts of apparent temperature exposures on asthmatic children might be modified by sex. However, the result of the current modification analyses should be cautiously interpreted due to the limited sample size in this study. More studies are needed to further confirm this finding and explore the underlying biological mechanisms.

This study has some limitations. First, we assumed that the relationships between indoor and outdoor apparent temperatures were the same in participants’ homes and the other indoor microenvironments at the same time of the day. By using this assumption, we calculated the personal apparent temperature exposure in other indoor microenvironments without actual measurements. If this assumption is incorrect, relying on it may introduce systematic or random errors in personal apparent temperature assessment. Second, the current study has the limitation of investigating how high average apparent temperature exposures (i.e., > 30°C) affect asthma, because this study was conducted in the early spring season. On the contrary, it could be a strength to mitigate the confounding effects caused by seasonal variations. Third, this study was conducted when indoor heating or cooling was rarely used in participants’ houses, when there was a high correlation between personal apparent temperature and ambient temperature. If the indoor-outdoor temperature difference was larger, the effect size of personal apparent temperature exposure may be different from the ones observed in the present study. More studies are suggested to explore how indoor air conditioning (heating or cooling) affects personal apparent temperature exposures and the associated health impacts in people with asthma.

## 5. Conclusion

Lower 1-week and 2-week average and minimal personal apparent temperature exposures and temperature variability were significantly associated with lower total and individual C-ACT scores reported by children with asthma. Our results support that, compared to ambient temperature exposure, personal apparent temperature exposure is potentially a stronger driver for the variability in asthma symptom scores. Maintaining a proper personal apparent temperature exposure could be an effective strategy for personalized asthma management.

## Supporting information

S1 Fig Schematic depicting the entire approach to calculate personal exposures to apparent temperature and apparent temperature exposure variability. (TIF)

S2 Fig The change in total C-ACT score associated with a 10°C lower in personal apparent temperature exposures and TV: Stratified by sex (21 males and 16 females). (TIF)

S3 Fig The change in C-ACT scores associated with a 10°C lower in personal apparent temperature exposures or a 10°C higher in TV: Excluding 7 measurements from 6 participants who had asthma exacerbation during the 2-week prior to the clinical visit. (TIF)

S1 Table The questions in the C-ACT questionnaire. (DOCX)

S2 Table Equations to calculate apparent temperature (T = temperature, RH = relative humidity). (DOCX)

S3 Table Personal air pollutant exposure. (DOCX)

S4 Table The model results of Fig 1. (DOCX)

S5 Table The model results of Fig 2. (DOCX)

S6 Table Repeated measures correlations coefficients. (DOCX)

S7 Table The interactions between sex and personal apparent temperature exposures. (DOCX)

S1 Appendix Statistical analysis. (DOCX)

S2 Appendix The minimal data set. (CSV)

S1 Graphical abstract (TIFF)
