# Supplementary figures and images for: Associations between personal apparent temperature exposures and asthma symptoms in children with asthma
Source: PLoS One. 2023 Nov 13;18(11):e0293603. doi: 10.1371/journal.pone.0293603 (PMC10642815; doi:10.1371/journal.pone.0293603)

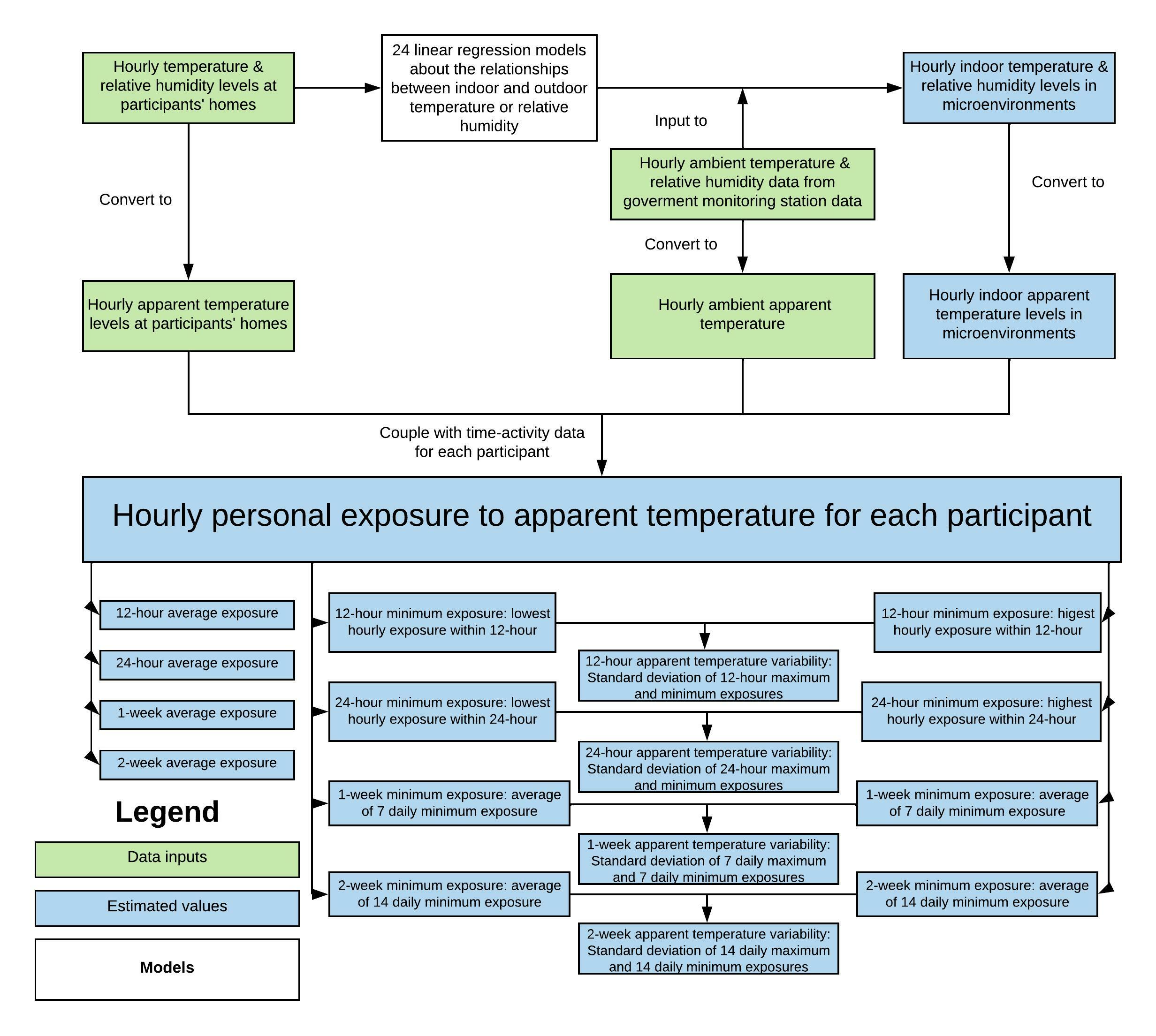

Supplement: S1 Fig — (TIF) [file pone.0293603.s001.tif]

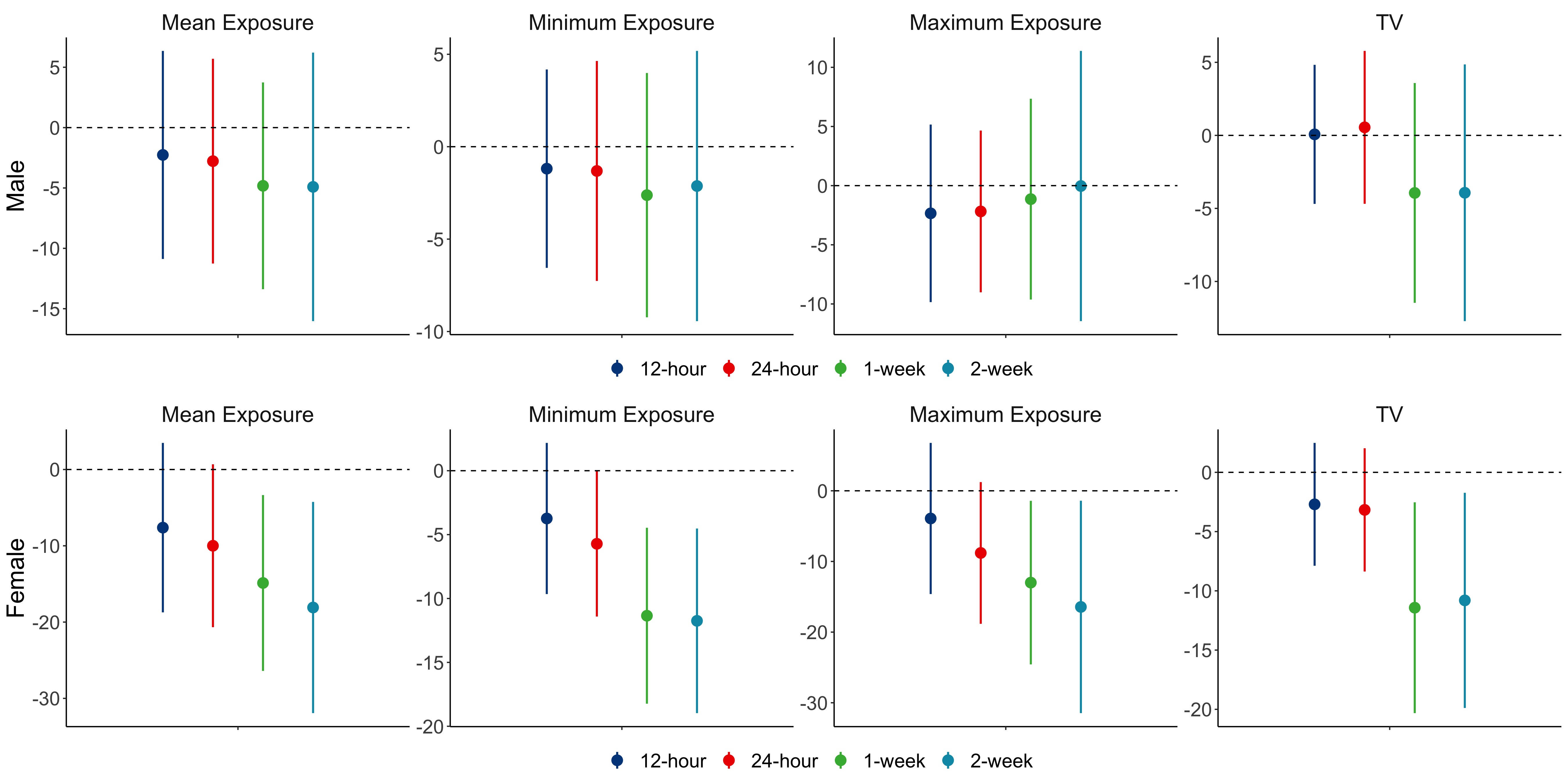

Supplement: S2 Fig — (TIF) [file pone.0293603.s002.tif]

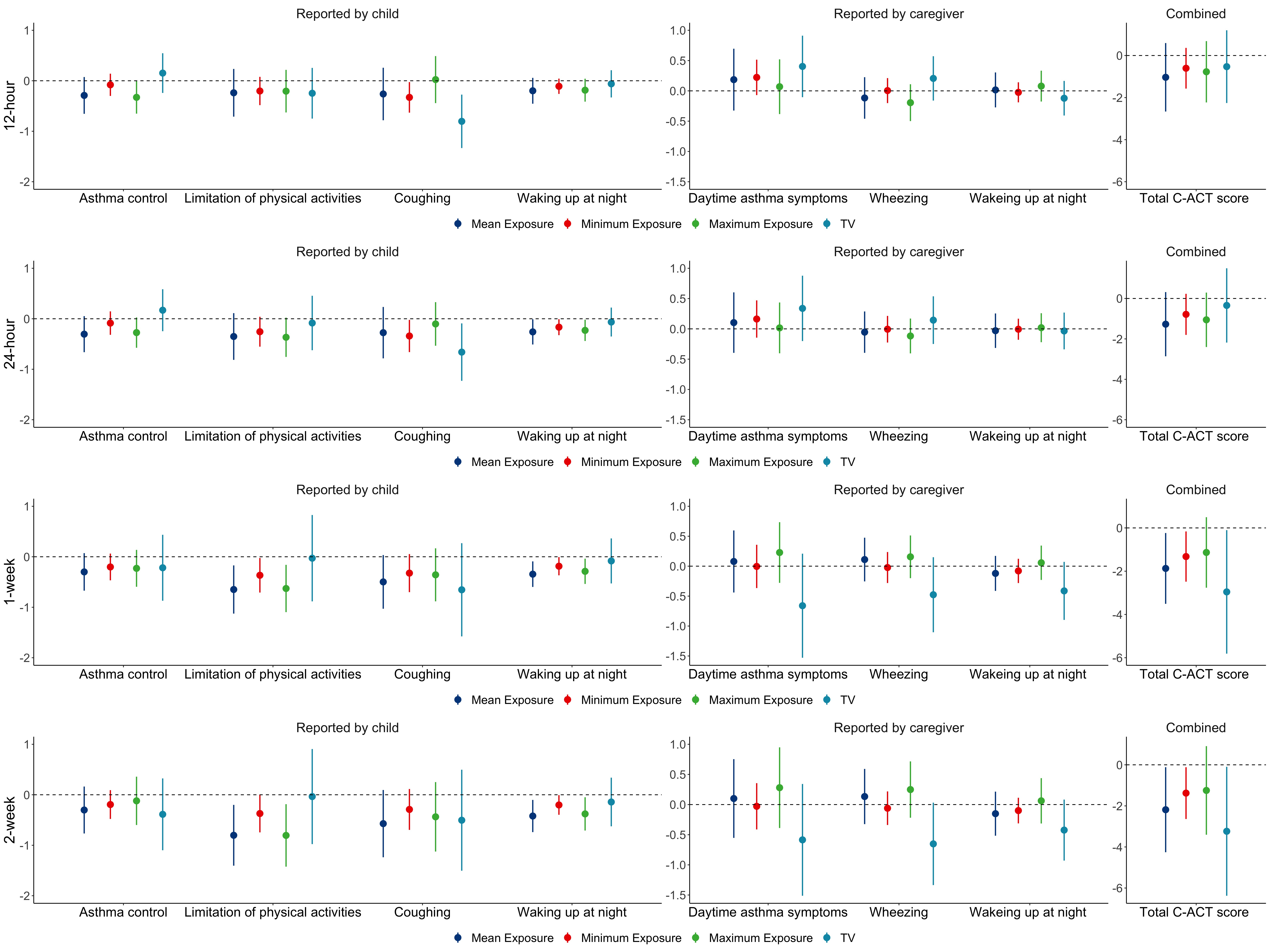

Supplement: S3 Fig — (TIF) [file pone.0293603.s003.tif]

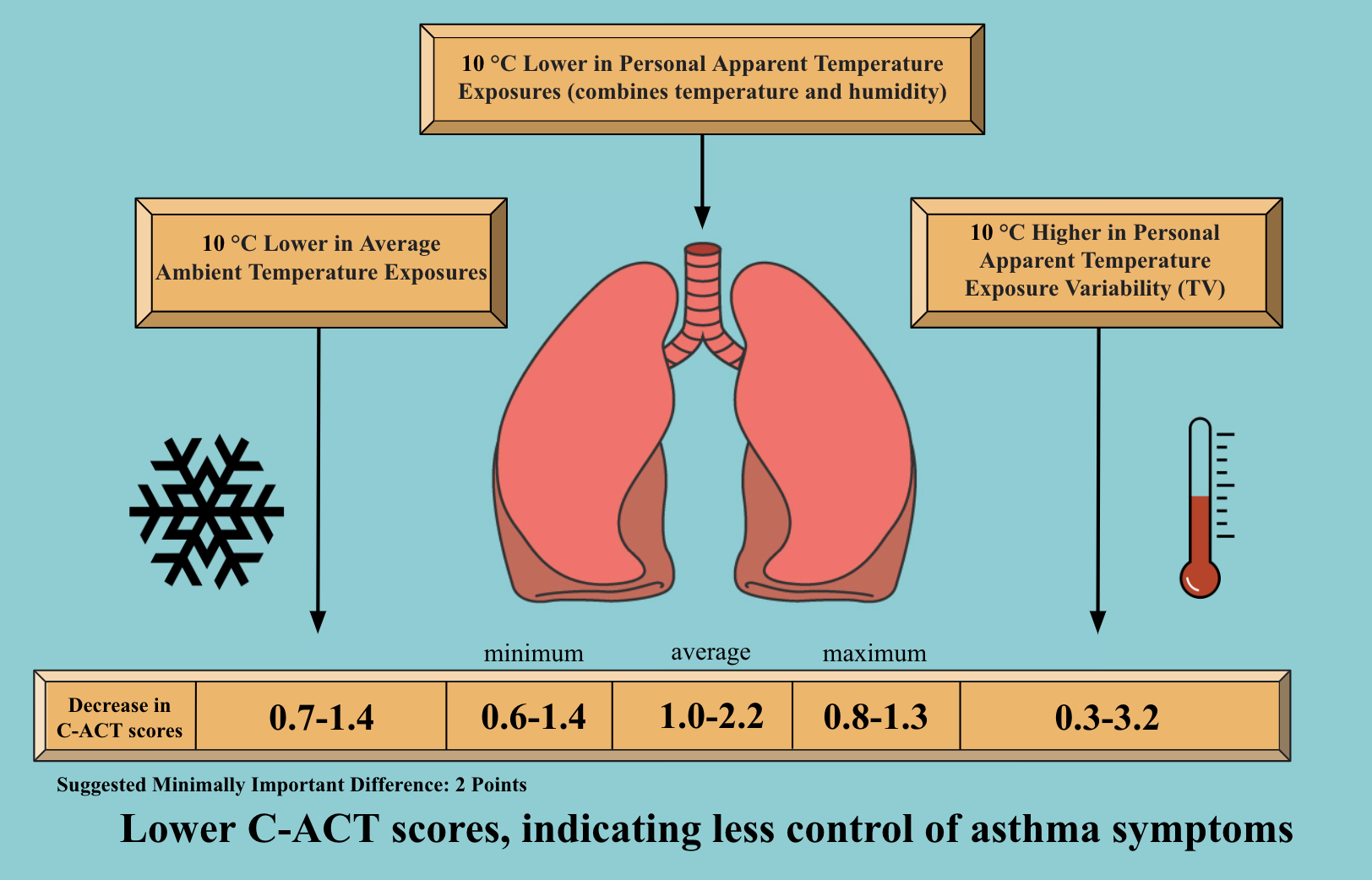

Supplement: S1 Graphical abstract — (TIFF) [file pone.0293603.s013.tiff]
