# Supplementary material for: Associations between personal apparent temperature exposures and asthma symptoms in children with asthma
Source: PLoS One. 2023 Nov 13;18(11):e0293603. doi: 10.1371/journal.pone.0293603 (PMC10642815; doi:10.1371/journal.pone.0293603)
Supplement: S1 Table — (DOCX) [file pone.0293603.s004.docx]

**S1 Table. The questions in the C-ACT questionnaire**

| **Answered by child with asthma (scores: 0: Very bad, 1: Bad, 2: Good, 3: Very good)** | |
| --- | --- |
| 1 | [**Asthma control**] How is your asthma today? |
| 2 | [**Limitation of physical activities**] How much of a problem is your asthma when you run, exercise or play sports? |
| 3 | [**Coughing**] Do you cough because of your asthma? |
| 4 | [**Waking up at night**] Do you wake up during the night because of your asthma? |
| **Answered by caregiver. (scores: 0: Everyday, 1: 19-24 days/month, 2: 11-18 days/month, 3: 4-10 days/month, 4: 1-3 days/month, 5: Not at all)** | |
| 5 | [**Daytime asthma symptoms**] During the last 4 weeks, on average, how many days per month did your child have any daytime asthma symptoms? |
| 6 | [**Wheezing**] During the last 4 weeks, how many days per month did your child wheeze during the day because of asthma? |
| 7 | [**Waking up at night**] During the last 4 weeks, on average, how many days per month did your child wake up during the night because of asthma? |
