# Supplementary material for: Associations between personal apparent temperature exposures and asthma symptoms in children with asthma
Source: PLoS One. 2023 Nov 13;18(11):e0293603. doi: 10.1371/journal.pone.0293603 (PMC10642815; doi:10.1371/journal.pone.0293603)
Supplement: S2 Table — (DOCX) [file pone.0293603.s005.docx]

**S2 Table. Equations to calculate apparent temperature** (T=temperature, RH=relative humidity)

|  |  | Equation | Comments |
| --- | --- | --- | --- |
| Step 1 | Steadman equation | Apparent temperature = 0.5 * {T + 61.0 + [(T-68.0)*1.2] + (RH*0.094)} | If this result is 80 degrees F or higher, starting step 2. |
| Step 2 | Rothfusz  equation | HI = -42.379 + 2.04901523*T + 10.14333127*RH - .22475541*T*RH - .00683783*T*T - .05481717*RH*RH + .00122874*T*T*RH + .00085282*T*RH*RH - .00000199*T*T*RH*RH | If the RH is less than 13% and the temperature is between 80 and 112 degrees F, follow Step 3a.  If the RH is greater than 85% and the temperature is between 80 and 87 degrees F, follow Step 3b. |
| Step 3a | Rothfusz  equation | Step 2 result - [(13-RH)/4]*SQRT{[17-ABS(T-95.)]/17} |  |
| Step 3b | Rothfusz  equation | Step 2 result + [(RH-85)/10] * [(87-T)/5] |  |
