# Supplementary material for: Associations between personal apparent temperature exposures and asthma symptoms in children with asthma
Source: PLoS One. 2023 Nov 13;18(11):e0293603. doi: 10.1371/journal.pone.0293603 (PMC10642815; doi:10.1371/journal.pone.0293603)
Supplement: S3 Table — (DOCX) [file pone.0293603.s006.docx]

**S3 Table.** **Personal air pollutant exposure**

|  | **Mean ± SD** | **Median [IQR]** | **Range** |
| --- | --- | --- | --- |
| **PM_2.5_** (μg/m^3^, 24-hour average) | | | |
| 12-hour | 40.1 ± 18.7 | 37.6 [23.8] | 2.0 – 81.6 |
| 24-hour | 39.0 ± 17.0 | 36.0 [23.7] | 9.0 – 86.1 |
| 1-week | 36.5 ± 13.7 | 35.1 [21.7] | 10.8 – 65.3 |
| 2-week | 36.7 ± 12.5 | 36.2 [20.8] | 8.8 – 62.3 |
| **O_3_** (ppb, 24-hour average) | | | |
| 12-hour | 14.2 ± 6.5 | 13.2 [9.2] | 4.5 – 34.8 |
| 24-hour | 14.0 ± 5.1 | 13.7 [6.3] | 5.2 – 30.6 |
| 1-week | 14.2 ± 3.5 | 13.9 [4.7] | 6.9 – 27.1 |
| 2-week | 13.9 ± 3.2 | 13.9 [4.0] | 7.8 – 27.9 |
