# Supplementary material for: Associations between personal apparent temperature exposures and asthma symptoms in children with asthma
Source: PLoS One. 2023 Nov 13;18(11):e0293603. doi: 10.1371/journal.pone.0293603 (PMC10642815; doi:10.1371/journal.pone.0293603)
Supplement: S4 Table — (DOCX) [file pone.0293603.s007.docx]

**S4 Table**. The model results of **Fig 1**.

|  | C-ACT Scores |  | Average Apparent Temperature Exposure | | Minimum Apparent Temperature Exposure | | Maximum Apparent Temperature Exposure | | Apparent Temperature Exposure Variability | |
| --- | --- | --- | --- | --- | --- | --- | --- | --- | --- | --- |
|  |  |  | Effect size | P value | Effect size | P value | Effect size | P value | Effect size | P value |
| Reported by Child | Asthma control | 12-hour | -0.29 | 0.117 | -0.08 | 0.473 | -0.33 | 0.046 | 0.15 | 0.445 |
|  | Asthma control | 24-hour | -0.30 | 0.093 | -0.09 | 0.466 | -0.27 | 0.073 | 0.17 | 0.421 |
|  | Asthma control | 1-week | -0.30 | 0.113 | -0.20 | 0.134 | -0.23 | 0.217 | -0.22 | 0.510 |
|  | Asthma control | 2-week | -0.30 | 0.201 | -0.19 | 0.182 | -0.12 | 0.619 | -0.39 | 0.283 |
|  | Limitation of physical activities | 12-hour | -0.24 | 0.320 | -0.20 | 0.155 | -0.21 | 0.336 | -0.25 | 0.332 |
|  | Limitation of physical activities | 24-hour | -0.35 | 0.135 | -0.26 | 0.089 | -0.37 | 0.065 | -0.08 | 0.759 |
|  | Limitation of physical activities | 1-week | -0.65 | 0.008 | -0.37 | 0.036 | -0.63 | 0.009 | -0.03 | 0.948 |
|  | Limitation of physical activities | 2-week | -0.80 | 0.009 | -0.37 | 0.051 | -0.81 | 0.011 | -0.04 | 0.941 |
|  | Coughing | 12-hour | -0.26 | 0.320 | -0.33 | 0.033 | 0.02 | 0.921 | -0.80 | 0.003 |
|  | Coughing | 24-hour | -0.28 | 0.286 | -0.34 | 0.036 | -0.10 | 0.638 | -0.66 | 0.023 |
|  | Coughing | 1-week | -0.50 | 0.066 | -0.32 | 0.091 | -0.36 | 0.180 | -0.65 | 0.163 |
|  | Coughing | 2-week | -0.57 | 0.092 | -0.29 | 0.158 | -0.44 | 0.211 | -0.50 | 0.320 |
|  | Waking up at night | 12-hour | -0.20 | 0.128 | -0.11 | 0.157 | -0.19 | 0.107 | -0.06 | 0.656 |
|  | Waking up at night | 24-hour | -0.26 | 0.042 | -0.17 | 0.040 | -0.23 | 0.033 | -0.06 | 0.655 |
|  | Waking up at night | 1-week | -0.34 | 0.008 | -0.19 | 0.043 | -0.29 | 0.024 | -0.08 | 0.715 |
|  | Waking up at night | 2-week | -0.42 | 0.010 | -0.20 | 0.042 | -0.38 | 0.025 | -0.14 | 0.557 |
| Reported by caregiver | Daytime asthma symptoms | 12-hour | 0.19 | 0.473 | 0.22 | 0.134 | 0.07 | 0.764 | 0.40 | 0.118 |
|  | Daytime asthma symptoms | 24-hour | 0.10 | 0.682 | 0.16 | 0.297 | 0.02 | 0.939 | 0.34 | 0.217 |
|  | Daytime asthma symptoms | 1-week | 0.08 | 0.765 | 0.00 | 0.981 | 0.23 | 0.374 | -0.66 | 0.135 |
|  | Daytime asthma symptoms | 2-week | 0.10 | 0.761 | -0.03 | 0.883 | 0.28 | 0.409 | -0.59 | 0.213 |
|  | Wheezing | 12-hour | -0.12 | 0.500 | 0.00 | 0.967 | -0.19 | 0.207 | 0.21 | 0.268 |
|  | Wheezing | 24-hour | -0.05 | 0.754 | -0.01 | 0.959 | -0.12 | 0.420 | 0.14 | 0.470 |
|  | Wheezing | 1-week | 0.11 | 0.550 | -0.02 | 0.863 | 0.16 | 0.388 | -0.48 | 0.134 |
|  | Wheezing | 2-week | 0.13 | 0.565 | -0.06 | 0.668 | 0.25 | 0.293 | -0.65 | 0.061 |
|  | Waking up at night | 12-hour | 0.02 | 0.915 | -0.02 | 0.771 | 0.08 | 0.543 | -0.12 | 0.401 |
|  | Waking up at night | 24-hour | -0.03 | 0.833 | -0.01 | 0.947 | 0.02 | 0.873 | -0.03 | 0.822 |
|  | Waking up at night | 1-week | -0.12 | 0.414 | -0.08 | 0.440 | 0.06 | 0.695 | -0.41 | 0.094 |
|  | Waking up at night | 2-week | -0.15 | 0.415 | -0.10 | 0.355 | 0.06 | 0.744 | -0.42 | 0.100 |
|  | Total C-ACT score | 12-hour | -1.04 | 0.210 | -0.60 | 0.218 | -0.77 | 0.296 | -0.53 | 0.546 |
|  | Total C-ACT score | 24-hour | -1.27 | 0.115 | -0.79 | 0.128 | -1.06 | 0.123 | -0.34 | 0.712 |
|  | Total C-ACT score | 1-week | -1.87 | 0.025 | -1.32 | 0.026 | -1.13 | 0.171 | -2.96 | 0.042 |
|  | Total C-ACT score | 2-week | -2.19 | 0.039 | -1.38 | 0.032 | -1.25 | 0.254 | -3.24 | 0.043 |

Note: The effect size indicates the percent change in C-ACT scores associated with 10 °C lower in personal apparent temperature exposure or 10°C higher in personal apparent temperature exposure variability.
