# Supplementary material for: Associations between personal apparent temperature exposures and asthma symptoms in children with asthma
Source: PLoS One. 2023 Nov 13;18(11):e0293603. doi: 10.1371/journal.pone.0293603 (PMC10642815; doi:10.1371/journal.pone.0293603)
Supplement: S5 Table — (DOCX) [file pone.0293603.s008.docx]

**S5 Table**. **The model results of Fig 2**

|  | C-ACT Scores |  | Average Ambient Temperature | |
| --- | --- | --- | --- | --- |
|  |  |  | Effect size | P value |
| Reported by Child | Asthma control | 12-hour | -0.106 | 0.396 |
|  | Asthma control | 24-hour | -0.149 | 0.218 |
|  | Asthma control | 1-week | -0.257 | 0.035 |
|  | Asthma control | 2-week | -0.249 | 0.100 |
|  | Limitation of physical activities | 12-hour | -0.255 | 0.115 |
|  | Limitation of physical activities | 24-hour | -0.340 | 0.031 |
|  | Limitation of physical activities | 1-week | -0.421 | 0.008 |
|  | Limitation of physical activities | 2-week | -0.539 | 0.008 |
|  | Coughing | 12-hour | -0.222 | 0.210 |
|  | Coughing | 24-hour | -0.278 | 0.108 |
|  | Coughing | 1-week | -0.357 | 0.042 |
|  | Coughing | 2-week | -0.396 | 0.076 |
|  | Waking up at night | 12-hour | -0.141 | 0.108 |
|  | Waking up at night | 24-hour | -0.176 | 0.040 |
|  | Waking up at night | 1-week | -0.189 | 0.026 |
|  | Waking up at night | 2-week | -0.239 | 0.027 |
| Reported by caregiver | Daytime asthma symptoms | 12-hour | 0.088 | 0.602 |
|  | Daytime asthma symptoms | 24-hour | 0.087 | 0.599 |
|  | Daytime asthma symptoms | 1-week | 0.031 | 0.854 |
|  | Daytime asthma symptoms | 2-week | 0.062 | 0.774 |
|  | Wheezing | 12-hour | -0.065 | 0.578 |
|  | Wheezing | 24-hour | -0.032 | 0.779 |
|  | Wheezing | 1-week | 0.071 | 0.551 |
|  | Wheezing | 2-week | 0.101 | 0.508 |
|  | Waking up at night | 12-hour | 0.031 | 0.740 |
|  | Waking up at night | 24-hour | 0.028 | 0.765 |
|  | Waking up at night | 1-week | -0.018 | 0.846 |
|  | Waking up at night | 2-week | -0.043 | 0.725 |
|  | Total C-ACT score | 12-hour | -0.721 | 0.188 |
|  | Total C-ACT score | 24-hour | -0.911 | 0.089 |
|  | Total C-ACT score | 1-week | -1.246 | 0.020 |
|  | Total C-ACT score | 2-week | -1.406 | 0.042 |

Note: The effect size indicates the percent change in C-ACT scores associated with 10 °C lower in average ambient temperature exposure.
