# Supplementary material for: Associations between personal apparent temperature exposures and asthma symptoms in children with asthma
Source: PLoS One. 2023 Nov 13;18(11):e0293603. doi: 10.1371/journal.pone.0293603 (PMC10642815; doi:10.1371/journal.pone.0293603)
Supplement: S6 Table — (DOCX) [file pone.0293603.s009.docx]

**S6 Table**. **Repeated measures correlations coefficients**

| 12-hour Personal Exposure | Mean | Minimum | Maximum | TV |
| --- | --- | --- | --- | --- |
| Minimum | 0.86 | 1 |  |  |
| Maximum | 0.88 | 0.69 | 1 |  |
| TV | -0.32 | -0.70 | 0.04 | 1 |
| Ambient Mean Temperature | 0.84 | 0.80 | 0.79 | -0.32 |

| 24-hour Personal Exposure | Mean | Minimum | Maximum | TV |
| --- | --- | --- | --- | --- |
| Minimum | 0.85 | 1 |  |  |
| Maximum | 0.84 | 0.69 | 1 |  |
| TV | -0.23 | -0.59 | 0.17 | 1 |
| Ambient Mean Temperature | 0.91 | 0.84 | 0.81 | 0.24 |

| 1-week Personal Exposure | Mean | Minimum | Maximum | TV |
| --- | --- | --- | --- | --- |
| Minimum | 0.86 | 1 |  |  |
| Maximum | 0.91 | 0.76 | 1 |  |
| TV | -0.21 | -0.58 | 0.20 | 1 |
| Ambient Mean Temperature | 0.94 | 0.87 | 0.88 | -0.07 |

| 2-week Personal Exposure | Mean | Minimum | Maximum | TV |
| --- | --- | --- | --- | --- |
| Minimum | 0.87 | 1 |  |  |
| Maximum | 0.90 | 0.75 | 1 |  |
| TV | -0.24 | -0.61 | 0.05 | 1 |
| Ambient Mean Temperature | 0.92 | 0.87 | 0.88 | -0.26 |
