# Supplementary material for: Associations between personal apparent temperature exposures and asthma symptoms in children with asthma
Source: PLoS One. 2023 Nov 13;18(11):e0293603. doi: 10.1371/journal.pone.0293603 (PMC10642815; doi:10.1371/journal.pone.0293603)
Supplement: S7 Table — (DOCX) [file pone.0293603.s010.docx]

**S7 Table.** **The interactions between sex and personal apparent temperature exposures**

|  | Sex × Average Apparent Temperature Exposure | | Sex × Minimum Apparent Temperature Exposure | | Sex × Maximum Apparent Temperature Exposure | | Sex × Apparent Temperature Exposure Variability | |
| --- | --- | --- | --- | --- | --- | --- | --- | --- |
|  | B | p-value | B | p-value | B | p-value | B | p-value |
| 12-hour | -0.17 | 0.27 | -0.11 | 0.24 | -0.08 | 0.59 | 0.21 | 0.23 |
| 24-hour | -0.21 | 0.20 | -0.15 | 0.14 | -0.19 | 0.18 | 0.24 | 0.20 |
| 1-week | -0.29 | 0.09 | -0.23 | 0.052 | -0.32 | 0.07 | 0.39 | 0.19 |
| 2-week | -0.18 | 0.28 | **-0.27** | **0.037** | **-0.46** | **0.048** | 0.43 | 0.18 |
