# Supplementary material for: Associations between personal apparent temperature exposures and asthma symptoms in children with asthma
Source: PLoS One. 2023 Nov 13;18(11):e0293603. doi: 10.1371/journal.pone.0293603 (PMC10642815; doi:10.1371/journal.pone.0293603)
Supplement: S1 Appendix — (DOCX) [file pone.0293603.s011.docx]

**Statistical analysis**

Linear mixed-effect regression (LMER) models were constructed to examine the cross-sectional association of C-ACT scores with average, minimum, and maximum personal apparent temperature exposure and apparent temperature exposure variability (TV) following ***Formula 1***. LMER models were used to examine the cross-sectional associations between C-ACT scores and ambient temperature exposure following ***Formula 2***. From the model output, we calculated percent change (and 95% confidence interval) of the C-ACT scores associated with 10 ^◦^C increase in personal apparent temperature exposure.

To investigate whether the associations between total C-ACT scores and personal apparent temperature exposures were modified by sex, we assessed the interaction between sex and apparent temperature exposures in LMER models following ***Formula 3*.**

***Formula 1***

$${C-ACT}_{ij}\sim\beta_{0}+\beta_{1}{Apparent Temperature}_{ij}+\beta_{2}{PM2.5}_{ij}+\beta_{3}{O3}_{ij}+\beta_{4}{EOS}_{i}+\beta_{5}{Fever}_{ij}+\beta_{6}{ICS}_{ij}+\beta_{7}{Flare}_{ij}+\beta_{8}{Sex}_{i}+\beta_{9}{Age}_{i}+P_{i}\boldsymbol{+Ɛ}$$

***Formula 2***

$${C-ACT}_{ij}\sim\beta_{0}+\beta_{1}{Ambient Temperature}_{ij}+\beta_{2}{PM2.5}_{ij}+\beta_{3}{O3}_{ij}+\beta_{4}{EOS}_{i}+\beta_{5}{Fever}_{ij}+\beta_{6}{ICS}_{ij}+\beta_{7}{Flare}_{ij}+\beta_{8}{RH}_{ij}+\beta_{9}{Sex}_{i}+\beta_{10}{Age}_{i}+P_{i}\boldsymbol{+Ɛ}$$

***Formula 3***

$${C-ACT}_{ij}\sim\beta_{0}+\beta_{1}{Apparent Temperature}_{ij}+\beta_{2}{PM2.5}_{ij}+\beta_{3}{O3}_{ij}+\beta_{4}{EOS}_{i}+\beta_{5}{Fever}_{ij}+\beta_{6}{ICS}_{ij}+\beta_{7}{Flare}_{ij}+\beta_{8}{Sex}_{i}+\beta_{9}{Age}_{i}+\beta_{10}{Sex}_{i}{*Apparent Temperature}_{ij}+P_{i}\boldsymbol{+Ɛ}$$

***Codebook***

$i:$ participant id number. ($i$= 1, 2, …, 43)

$j$: sample number ($j$= 1, 2, 3, 4)

${C-ACT}_{ij}$: Individual or total C-ACT scores.

${Apparent Temperature}_{ij}$: average, minimum, or maximum personal apparent temperature exposures or temperature variability over the 12-hours, 24-hours, 1-week, or 2-weeks prior to C-ACT measurement. (unit: ^◦^C)

${Ambient Temperature}_{ij}$: average ambient temperature exposures over the 12-hours, 24-hours, 1-week, or 2-weeks prior to C-ACT measurement. (unit: ^◦^C)

${RH}_{ij}$: ambient relative humidity measured averaged over the same period of ambient temperature exposures. (unit: %)

${EOS}_{i}$: baseline eosinophil number. (unit: /µL)

${Fever}_{ij}$: upper respiratory tract infection like symptoms status during the 2 weeks prior to the clinical visit. (0=no respiratory infection symptoms, 1=respiratory infection symptoms)

${ICS}_{ij}$: inhaled corticosteroids usage status during the 2 weeks prior to the clinical visit. (0=not used, 1=used)

${Flare}_{ij}$: the status of asthma exacerbation during the 2 weeks prior to the clinical visit (0=no exacerbation, 1=exacerbation)

${PM2.5}_{ij}$: personal PM_2.5_ exposure averaged over the same period of temperature exposures (unit: µg/m^3^)

${O3}_{ij}$: personal O_3_ exposure averaged over the same period of temperature exposures (unit: ppb)

$P_{i}$: individual-specific random intercept

$Ɛ$: residual
